# A Fast Universal Immobilization of Immunoglobulin G at 4°C for the Development of Array-based Immunoassays

**DOI:** 10.1371/journal.pone.0051370

**Published:** 2012-12-07

**Authors:** Shu-Lin Guo, Po-Chung Chen, Ming-Shuo Chen, Yu-Che Cheng, Jun-Mu Lin, Hoong-Chien Lee, Chien-Sheng Chen

**Affiliations:** 1 Graduate Institute of Systems Biology and Bioinformatics, National Central University, Jhongli, Taiwan; 2 Department of Anesthesiology, Cathay General Hospital, Taipei, Taiwan; 3 Department of Medical Research, Cathay General Hospital, Taipei, Taiwan; 4 Institute of Biomedical Engineering, National Central University, Jhongli, Taiwan; University of Strathclyde, United Kingdom

## Abstract

To maintain the antibody activity and enhance performance of array-based immunoassays, protein G was used to allow a shorter duration of immunoglobulin G immobilization at 4°C, with the antibody placed in the appropriate orientation. The multiplexed detection of six pain-related message molecules (PRMMs) was used as examples for the development of array-based immunoassays: substance P, calcitonin gene-related peptide, nerve growth factor, brain-derived neurotrophic factor, tumor necrosis factor-α, and β-endorphin. Protein G- and non-protein G-coated slides were tested. Compared to non-protein G immunoassays, protein G shortened the antibody immobilization time at 4°C from overnight to 2 hours. Only protein G-facilitated immunoassays succeeded in simultaneously detecting all six PRMMs with high specificity. Dose-response curves showed that the limits of detection of the protein G-multiplexed immunoassays for the PRMMs was approximately 164, 167, 120, 60, 80, and 92 pg/ml, respectively. Thus, protein G effectively shortens the duration of antibody immobilization at 4°C, allowing the use of sensitive array-based immunoassays for the simultaneous detection of PRMMs.

## Introduction

Proteomics profiles the protein network, changes in protein abundance, protein interactions and posttranslational modifications in biological systems [1]–[5]. Assays for high-throughput large-scale quantification of protein concentrations and interactions are needed. Traditionally, two-dimensional gel electrophoresis with mass spectrometry identification was commonly used in proteomics research [6]–[10]. However, this technique requires intensive sample preparation, time-consuming processes, and specially trained technicians.

Array-based immunoassays are promising miniature analytical tools that allow simultaneous detection and quantification of antigens in a small sample. These tools offer rapid analysis for proteomics research, identification of biomarkers, and clinic diagnosis of diseases [11]–[14]. Antibody attachment strategies have a profound influence on array-based immunoassays [15], [16]. Aldehyde, epoxysilane, poly-L-lysine, and nitrocellulose are commonly used to modify glass slides for antibody immobilization. The immobilization duration is usually up to 12 hours at room temperature [17]. Although antibodies are relatively stable proteins, antibodies may become inactivated and degraded during long immobilizations at room temperature. Previous studies showed that the deamidation of antibodies is more successful at 25°C than at 5°C [18]. Therefore, immobilizing antibodies at low temperatures such as 4°C is ideal. However, the immobilization duration at 4°C is overnight, which increases the amount of laborious experimental work and the possibility of degradation.

Protein G is an immunoglobulin-binding protein that is expressed on the cell surface of group G Streptococcal bacteria. The most pronounced characteristic of protein G is its ability to universally bind the Fc region of most IgG subtypes with high affinity [19]. The use of protein G for improving the orientation of antibodies on chips has been studied [20], [21]. However, protein G-coated slides for facilitating shorter antibody immobilization durations at 4°C were not discussed.

Here, we developed a multi-well array-based immunoassays with the aid of protein G, and, for demonstration, applied it on the detection of six pain-related message molecules (PRMMs): substance P (SP), calcitonin-gene related peptide (CGRP), nerve growth factor (NGF), brain-derived neurotrophic factor (BDNF), tumor necrosis factor alpha (TNF-α), and beta-endorphin (β-endorphin). Each well had an array of 24 immobilized antibody spots. Among these proteins, SP [22] and CGRP [23] are pain-specific neurotransmitters involved in pain perception; NGF and BDNF are neurotrophic factors that may have important roles in the development of neuropathic pain [24]; TNF-α is regarded as an inflammatory cytokine that can interact with other neurotrophic factors and modify pain sensation [25]; Beta-endorphin is the most important natural suppressor of hyperexcitability [26]. The simultaneous detection of these six representative PRMMs will allow us to clarify the complex processes underlying the pain pathway for further studies. A multi-well slide platform was used to increase the sample throughput per slide, facilitating the generation of dose-response curves for the six PRMMs. The antibody immobilization time, specificity, limit of detection, dynamic range, and sensitivity of protein G facilitated array-based immunoassays were investigated.

## Results and Discussions

### Immobilization of PRMM Antibodies on Slides

The slide surfaces should prevent droplet solutions from spreading and allow for a consistent spot size, unified spot morphology, and condensed proteins in the printed arrays. Good spot morphology is indicated by identical printing conditions and by the quality of immobilization on the array surface. Our previous study evaluated and tested four commercial slides, including the FAST slide (Whatman, USA), the MaxiSorp microarray slide (Nunc, USA), aldehyde-derivatized slides (BaiO, China), and the Protein slide (FullMoon BioSystems, USA). Results indicated that aldehyde-derivatized slides provided the best signal performance and greatest uniform quality among the four commercial slides [27]; thus, aldehyde-derivatized slides were used in this study.

**Figure 1:**
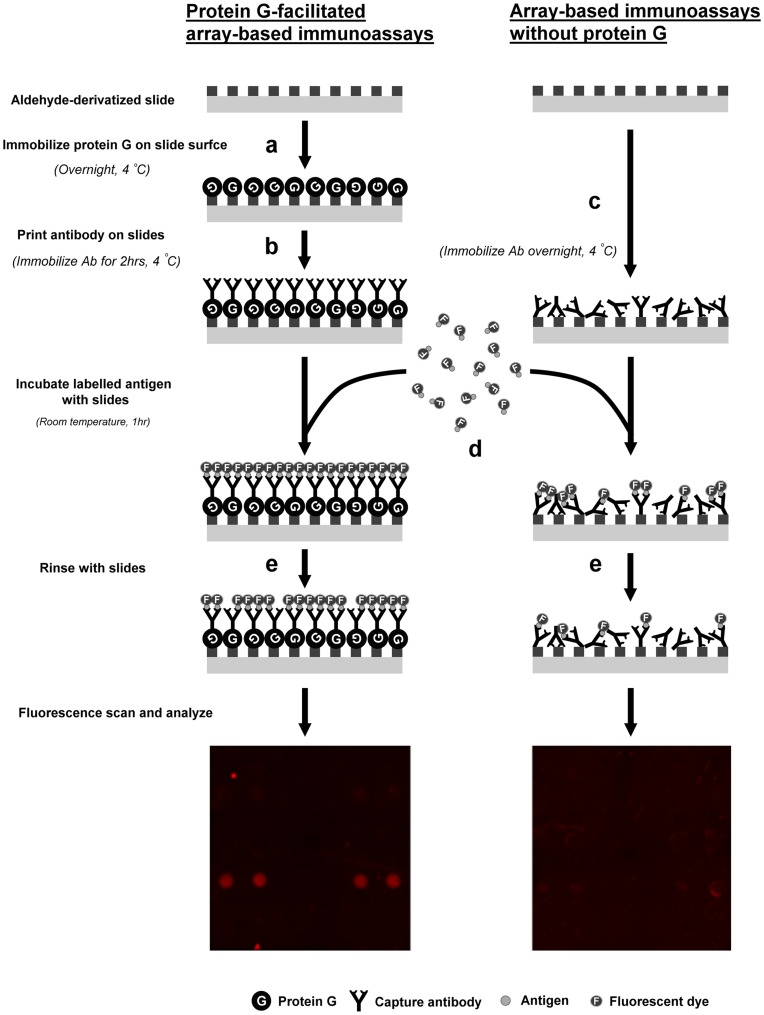
The PRMM IgG array fabrication with protein G or without protein G and array-based immunoassay procedures. Six PRMMs were directly labeled with fluorescent dye and probed with antibody slides (aldehyde-derivatized slides coated with or without protein G). Step a: The aldehyde-derivated slide was coated with protein G. Step b: The antibody was printed and immobilized on the protein G-coated slide at 4°C for 2 hours. Step c: The antibody was printed and immobilized on the aldehyde slide at 4°C for 14 hours. Step d: The samples were labeled with fluorescent dye and incubated with the antibody microarray slides at room temperature for 1 hour. Step e: The unbound samples were washed several times. Finally, the signals were directly detected using a fluorescence microarray scanner.

After testing the commercial slides, antibody immobilization was initiated at 4°C without protein G. The antibodies against the six PRMMs were printed directly onto aldehyde-derivatized slides and left overnight at 4°C to ensure covalent immobilization. We adopted the commonly used immunoassay format for proteomics research [28], [29], in which samples were labeled with fluorescent dye and probed with printed slides (Fig. 1). The labeled samples were incubated with the slides and reacted at room temperature for 1 hour. Thereafter, the unbound samples were washed. After carrying out the immunoassays, the fluorescence microarray scanner showed that the spots had inconsistent shapes and intensities, and several spots had no signal detection (Fig. 1). Protein G was used to modify the slide surface, which improved the array-based immunoassays and shortened the immobilization times. Protein G served as a universal strong surface-bound capture agent for the Fc fragments of various IgGs. The affinity between antibody and protein G is strong (equilibrium constant  =  10^9^∼10^10^M [30]); thus, antibodies can be immobilized in a short time, even at 4°C and in appropriate orientation with uniform face-out antigen-binding sites of antibodies. During the immobilization process, the printed antibodies were left on the protein G-coated slides for only 2 hours at 4°C. The array-based immunoassay was conductible immediately following the 2 hours of immobilization. The replicated spots showed consistent shapes and identical intensities, indicating that protein G facilitates array-based immunoassays.

### Cross Reactivity of the Array-based Immunoassays

**Figure 2:**
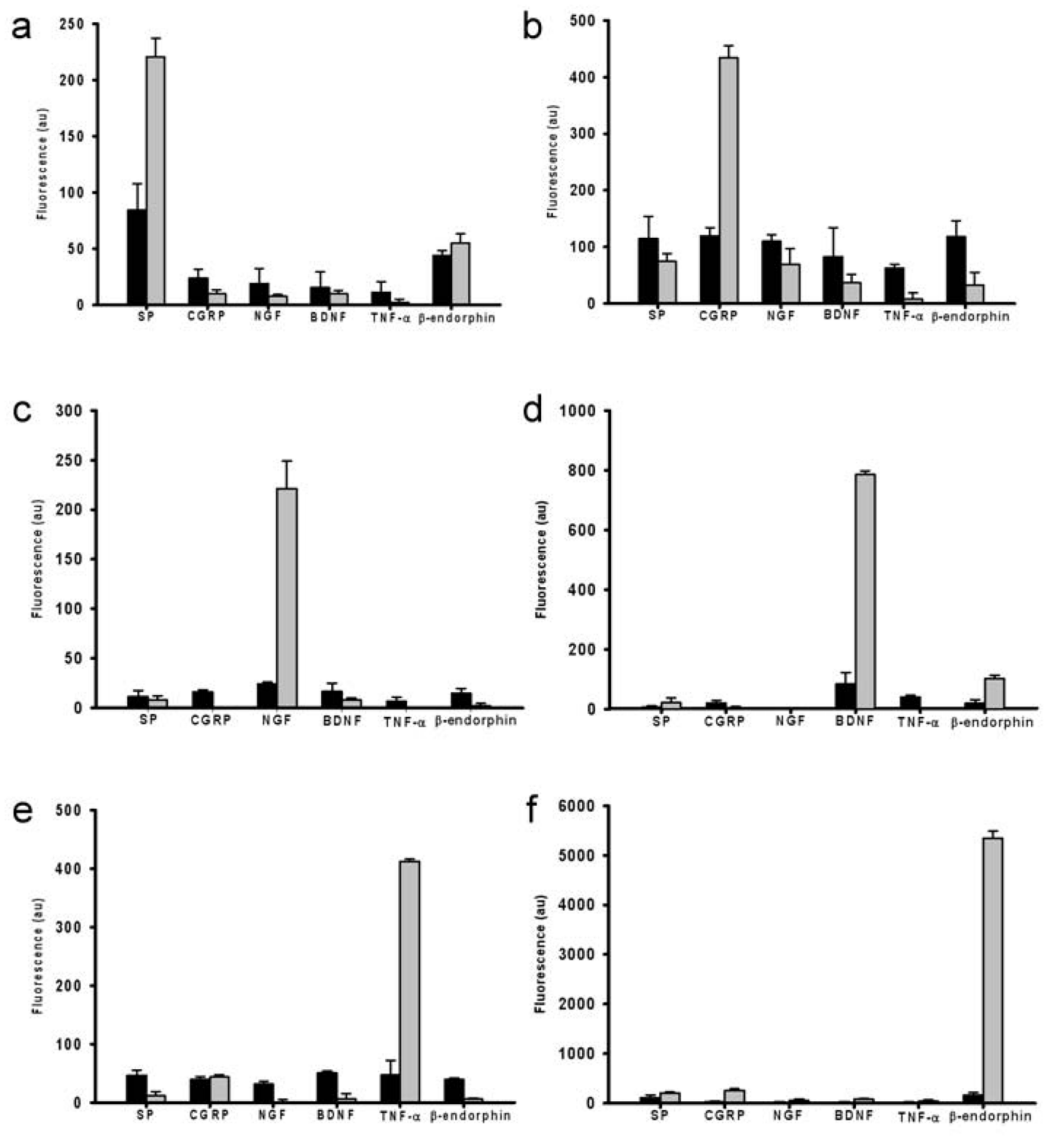
Cross-reactivity analysis of the array-based immunoassays with protein G (gray bar) and without protein G (black bar). Each single PRMM was individually probed with the IgG arrays containing all of the PRMM antibodies: (a) SP, (b) CGRP, (c) NGF, (d) BDNF, (e) TNF-α, and (f) β-endorphin. The error bars represent the standard deviations of four measurements.

Unlike conventional immunoassays such as ELISA, multiplexed slide-based assays may suffer from cross-reactivity due to possible nonspecific binding of capture antibodies [31]. To evaluate the cross-reactivity of the array-based immunoassays, each dye-labeled PRMM was applied to an individual antibody array, and the signals from the related antibody and non-related antibodies on both slides coated with and without protein G were measured. Fig. 2 shows the cross reactivity of the array-based immunoassays. Test results of immunoassay without protein G showed only an undistinguished signal for each PRMM (Fig. 2, black bar), suggesting that almost all of the signals were from non-specific bindings between the immobilized antibodies and PRMMs. Therefore, array-based immunoassays without protein G failed to detect PRMMs. In contrast, in tests with protein G, the intensity ratios of the related antibody to non-related antibody for detecting SP, CGRP, NGF, BDNF, TNF-α, and β-endorphin, were 13.06, 9.83, 59.0, 30.93, 29.46, and 42.24, respectively (Fig. 2, gray bar). These results indicated that this protein G-facilitated array-based immunoassay is able to detect PRMMs with high specificity. This improvement may result from the shorter antibody immobilization time, which allows for the preservation of the PRMM-binding ability. In additions, the improved antibody orientation may have improved the detection ability.

### Dose-response of each PRMM

Dose-response curves reveal the dynamic range, sensitivity, and detection limit of an immunoassay. Therefore, the dose response of each PRMM was further evaluated in a protein G-facilitated immunoassay. The six PRMMs were mixed and labeled with a DyLight 649 NHS ester in borate buffer. The mixed solution was series diluted with 1% w/v BSA in TBS. Each sample was added to an individual well and incubated at room temperature for 1 hour on an orbital shaker. Thereafter, the slides were washed three times, dried by brief centrifugation, and read using fluorescence microarray scanner at room temperature.

**Figure 3:**
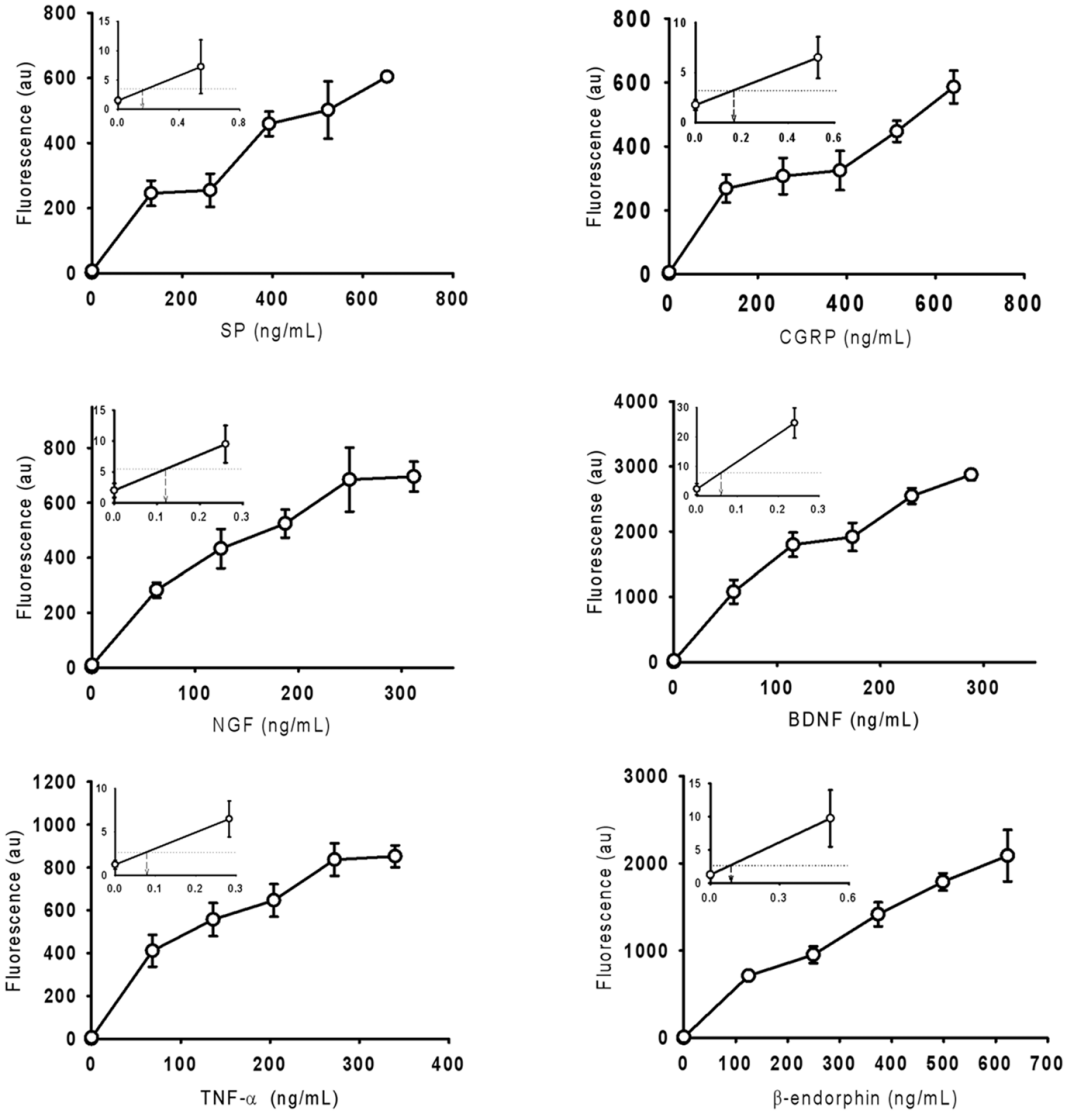
Dose–response curves of the six PRMMs in the array-based immunoassays with protein G. The inset-expanded curves show the location of the LOD. The straight horizontal line is three standard deviations higher than the mean intensity at zero concentration (negative control). The intersection of this line with the dose–response curve is the LOD, and is indicated by the vertical arrow. Error bars represent the standard deviations of four measurements.

The dose-response curves for the six PRMMs (Fig. 3) showed that fluorescent intensity increases with increased in PRMM concentration. The limit of detection (LOD) is defined as the lowest concentration of analyte that produces a fluorescent intensity three standard deviations higher than the mean intensity at a zero concentration (negative control). LODs for SP, CGRP, NGF, BDNF, TNF-α, and β-endorphin were determined to be approximately 164, 167, 120, 60, 80, 92 pg/mL, respectively (Table 1). These results indicated that the protein G-assisted array-based multiplexed immunoassay successfully detected PRMMs with good LOD values.

**Table 1:** Sensitivity, dynamic range and LOD for the detection of PRMMs using protein G-facilitated immunoassays.

Antigen	Sensitivity (au/ng/mL)	Dynamic range (ng/mL)	LOD (pg/mL)
SP	1.16	0.164–393	164
CGRP	0.91	0.167–641	167
BDNF	9.95	0.06–288	60
NGF	2.72	0.12–250	120
TNF-α	3.07	0.08–272	80
β-Endorphin	3.58	0.09–499	92

Note:

Dynamic range: from LOD to the “saturation point” (beyond which no significant signal increase was observed) of the dose-response curve.

Sensitivity: (the intensity of saturation point - LOD)/concentration difference.

The dynamic range of the PRMMs were also extracted from the dose-response curves (Fig. 3). BDNF, TNF-α, and β-Endorphin all had broad dynamic ranges of over 4 orders of magnitude (Fig. 3 and Table 1). Among the six PRMMs, NGF had the narrowest dynamic range, from 0.12 to 250 ng/mL (Table 1), which was still greater than 3 orders of magnitude.

Finally, the sensitivities may be determined from the slopes of the dose-response curves. All PRMMs showed steeper slopes at lower concentrations (Fig. 3), indicating high sensitivity in the low concentration range. BDNF showed the steepest slope with 18.24 au/ng/mL, in the range 0.24 ng/ml to 58 ng/ml. It was determined that the mean sensitivity for the six PRMMs, each averaged over its respective entire dynamic ranges, was 3.6 au/ng/mL, showing the high sensitivity of protein G-facilitated immunoassays.

In addition to having a shorter antibody immobilization time at 4°C, our protein G-facilitated immunoassays showed comparable performances with regard to LOD, dynamic range, and sensitivity (Table 1). Previous work on single detection of PRMM [32]–[38] reported LODs of several PRMMs similar to those achieved in this work. For example, the LOD for BDNF in this study was approximately 60 pg/mL, and the LOD for BDNF immunoassay reported by Nawa and coworker [32] was approximately 10 pg/mL. Furthermore, the LOD for TNF-α in this study was approximately 80 pg/mL, and the reported LOD for the TNF-α immunoassay was approximately 50 to 60 pg/mL [34], [35]. Although the LODs of our multiplexed immunoassays are not better than previous reports for single PRMM detection, our study is the first study to demonstrate simultaneous detection of six PRMMs with comparable dose responses.

## Conclusion

A multi-well array-based immunoassay for the quantitative and simultaneous detection of six important PRMMs was successfully developed in this study with the assistance of protein G. Protein G shortened the antibody immobilization duration at 4°C from overnight to 2 hours. LODs, dynamic ranges and sensitivities for all six PRMMs were determined from dose-response curves. We expect this technique of simultaneous detection of multiple PRMMs will further facilitate the understanding mechanisms of molecular pain.

## Materials and Methods

### Materials

Bovine serum albumin (BSA) was purchased from Sigma–Aldrich Co. (St. Louis, MO, USA). NaCl, Tris (base), and HCl were obtained from J.T. Baker (Phillipsburg, NJ, USA). Phosphate-buffered saline (PBS) and Tween 20 were purchased from One-Star Biotechnology (Taipei, Taiwan). Glycerol was obtained from Merck (Darmstadt, Germany). Recombinant protein G was purchased from BioVision (Mountain View, USA). DyLight 649 NHS Ester, Zeba™ desalt spin columns (0.5 mL), and sodium borate buffer (pH 8.5) were purchased from Pierce (Rockford, IL). Antibodies against NGF (rabbit polyclonal, whole antiserum), BDNF (monoclonal), TNF-α (monoclonal), β-endorphin (monoclonal), and SP (monoclonal) were purchased from Abcam (Cambridge, UK). An antibody against CGRP (rabbit polyclonal) was obtained from Santa Cruz (Santa Cruz, USA). Aldehyde-derivatized slides were provided by BaiO (Shanghai, China). Among the 6 chosen PRMMs, BDNF, NGF, TNF-α and β-endorphin were purchased from Abcam, SP was provided by GeneScript (Piscataway, USA), and CGRP was purchased from Calbiochem (Darmstadt, Germany). VP 110 washing buffer was purchased from V&P Scientific, Inc. (San Diego, USA).

### Fabrication of Protein G-facilitated IgG Arrays at 4°C

Aldehyde-derivatized glass slides were incubated overnight with 20 µg/mL of protein G in PBS with 1 mM EDTA at 4°C (Fig. 1a). All slides were washed three times for 5 minutes each with Tris-buffered saline including 0.05% v/v Tween 20 (TBST), rinsed with water, and dried by centrifugation prior to printing. The glass slide arrayer (V&P Scientific, Inc., USA) was used to print antibodies from their source plate at 4°C (Fig. 1b). Antibodies against SP, CGRP, NGF, BDNF, TNF-α, and β-endorphin were printed at concentrations of 100 µg/mL, 100 µg/mL, 3.25 mg/mL, 16.6 µg/mL, 50 µg/mL, and 100 µg/mL, respectively. The PBS with 50% v/v glycerol was used as a printing buffer. Four replicates of each antibody were printed on the slide surface. The spot diameter was approximately 500 µm, and the spacing between the spots was measured approximately 750 µm on the Y-axis and 1125 µm on the X-axis. The pins were washed on the arrayer with 1∶5 VP 110 washing buffer and 99% v/v EtOH between aspirates to prevent cross contamination. Eight identical arrays were printed on a protein G-coated slide and framed to the multi-wells. Thereafter, the arrays were refrigerated at 4°C for 2 hours before the immunoassays were performed. Following immobilization, the printed slides were washed three times in TBST for 5 minutes each to remove unbound antibodies. The slides were then ready for immunoassays.

### Fabrication of IgG Array without Protein G

The antibodies were printed on the aldehyde-derivatized glass slides as described above without protein G (Fig. 1c)., The antibodies were immobilized at 4°C overnight (14 hours) instead of 2 hours before performing the immunoassays to establish covalent linkage between the antibody and the slide surface. Other conditions and reagents were the same for the fabrication of protein G-facilitated IgG arrays.

### Sample Labeling

The six individual PRMMs were labeled with DyLight 649 NHS ester at a molar ratio of 1∶10 in 50 mM sodium borate buffer (pH 8.5) at room temperature for 1 hour (Fig. 1d). Thereafter, the labeling reactions were quenched with 1.67 M Tris-HCl by shaking at room temperature for 1 hour. The excess dye was removed using Zeba™ Desalt Spin columns.

### Immunoassays for Cross-reactivity Tests

The antibody printed slides were assembled with ProPlate frames (Grace Bio-Labs, Inc., USA) to form eight wells of identical antibody arrays, followed by a blocking with 3% w/v BSA at room temperature for 1 hour. Sixty microliters of each PRMM containing 1% w/v BSA was added to each individual well and incubated at room temperature for 1 hour on an orbital shaker. The concentrations of BDNF, NGF, TNF-α, β-endorphin, SP and CGRP were 208.3 ng/mL, 78 ng/mL, 2.6 µg/mL, 173.8 ng/mL, 5.8 µg/mL, and 1.3 µg/mL, respectively. After each well was washed with 250 µL of TBST three times, the frame was disassembled from the slide and then washed with TBST at room temperature for 10 minutes. Finally, each slide was washed with distilled water at room temperature three times for 10 minutes and briefly centrifuged until dry (Fig. 1e).

### Immunoassays for Dose-responses

The slides were framed and blocked following the procedure used for the cross-reactivity tests. Each standard PRMM was prepared at different concentrations to observe the dose response. Each sample contained all six PRMMs at the desired concentrations with 1% w/v BSA. The samples varied according to PRMM concentration. Each sample was added to an individual well and incubated at room temperature for 1 hour on an orbital shaker. After each well was washed with 250 µL of TBST three times, the frame was disassembled from the slide, and then the slide was washed with TBST at room temperature for 10 minutes. Finally, each slide was washed with distilled water at room temperature three times for 10 minutes and briefly centrifuged until dry (Fig. 1e). The negative control sample consisted of used 1% w/v BSA without PRMMs.

### Imaging and Data Analysis

An Axon GenePix 4000B (Molecular Devices, USA) was used to detect the signals at an excitation wavelength of 635 nm and an emission wavelength of 670 nm. All images were scanned at the same resolution of 10 µm. The valid spots were identified using GenePix Pro 6.0, and the final signal intensities were obtained by subtracting the background intensities from the spot intensities.
